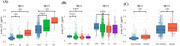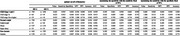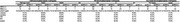# Optimizing Plasma *p*‐Tau217 Cutoffs for Amyloid Pathology According To Biological Factors

**DOI:** 10.1002/alz70856_102727

**Published:** 2025-12-24

**Authors:** Jihwan Yun, Daeun Shin, EUN HYE LEE, Jun Pyo Kim, Hongki Ham, Yuna Gu, Sung Hoon Kang, Hee Jin Kim, Duk L Na, Chi‐Hun Kim, Ko Woon Kim, Si Eun Kim, Yeshin Kim, Jaeho Kim, Na‐Yeon Jung, Yeo Jin Kim, Su Hyun Cho, Henrik Zetterberg, Kaj Blennow, Fernando Gonzalez‐Ortiz, Nicholas Ashton, Sang Won Seo, Hyemin Jang, K‐ROAD study groups

**Affiliations:** ^1^ Kyung Hee University Medical Center, Seoul, Korea, Republic of (South); ^2^ Samsung Medical Center, Gangnam‐gu, Seoul, Korea, Republic of (South); ^3^ Samsung Medical Center, Seoul, Korea, Republic of (South); ^4^ Samsung Medical Center, Sungkyunkwan University School of Medicine, Gangnam‐gu, Seoul, Korea, Republic of (South); ^5^ Korea University Guro Hospital, College of Medicine Korea University, Seoul, Korea, Republic of (South); ^6^ Happymind Clinic, Seoul, Korea, Republic of (South); ^7^ Hallym University Sacred Heart Hospital, Anyang, Gyeonggi‐do, Korea, Republic of (South); ^8^ Jeonbuk National University Medical School and Hospital, Jeonju, Korea, Republic of (South); ^9^ Inje University College of Medicine, Haeundae Paik Hospital, Busanjin‐gu, Busan, Korea, Republic of (South); ^10^ Kangwon National University, Chuncheon, Korea, Republic of (South); ^11^ Dongtan Sacred Heart Hospital, Hallym University College of Medicine, Hwaseong‐si, Gyeonggi‐do, Korea, Republic of (South); ^12^ Pusan National University Yangsan Hospital, Yangsan, Korea, Republic of (South); ^13^ Kangdong Sacred Heart Hospital, Seoul, Seoul, Korea, Republic of (South); ^14^ Chonnam National University Medical School, Gwangju, Korea, Republic of (South); ^15^ Sahlgrenska University Hospital, Mölndal, Sweden; ^16^ Clinical Neurochemistry Laboratory, Sahlgrenska University Hospital, Mölndal, Sweden; ^17^ Institute of Neuroscience and Physiology, University of Gothenburg, Mölndal, Sweden; ^18^ Department of Psychiatry and Neurochemistry, Institute of Neuroscience and Physiology, The Sahlgrenska Academy, University of Gothenburg, Mölndal, Sweden; ^19^ Seoul National University Hospital, Seoul National University College of Medicine, Jongno‐gu, Seoul, Korea, Republic of (South)

## Abstract

**Background:**

Plasma phosphorylated tau (*p*‐tau) 217 has shown strong performance in detecting β‐amyloid (Aβ) positivity on positron emission tomography (PET). However, kidney dysfunction, body mass index (BMI), and anemia are known to affect *p*‐tau217 levels. How the optimal cutoffs for *p*‐tau217 vary in relation to these factors has not been thoroughly explored.

**Method:**

In this multi‐center cross‐sectional study (2016–2023), 2,571 individuals with unimpaired cognition, mild cognitive impairment, or Alzheimer's‐type dementia underwent Aβ PET imaging, plasma *p*‐tau217 measurement (Alzpath Simoa immunoassay), and assessment for estimated glomerular filtration rate (eGFR), BMI, and anemia. Participants were categorized by eGFR (>60, 45–60, and <45 mL/min/1.73 m^2), BMI (underweight, normal weight, obesity, and super‐obesity), and anemia (anemia vs. non‐anemia). We identified optimal *p*‐tau217 cutoffs for detecting Aβ positivity (PET centiloid ≥25.5) and evaluated accuracy, sensitivity, and specificity in each subgroup.

**Result:**

Optimal *p*‐tau217 cutoffs were elevated in those with kidney dysfunction, underweight or obesity, and anemia compared to the reference groups. Specifically, optimal cutoffs for Aβ positivity were higher among individuals with eGFR <60 (0.635 for eGFR 45–60; 0.935 for eGFR <45) than among those with eGFR >60 (0.425). When the reference cutoff (0.425) was applied in the eGFR <45 group, diagnostic accuracy dropped to 48.3%. Underweight and anemia groups also showed increased optimal cutoffs (0.620 and 0.595) relative to the reference group (0.425), though using the reference cutoff slightly lowered specificity in these groups.

**Conclusion:**

These findings emphasize the importance of tailoring plasma *p*‐tau217 cutoffs based on kidney function, BMI, and anemia status to enhance diagnostic accuracy, especially in populations with more severe kidney dysfunction (eGFR <45).